# Microglia in frontotemporal lobar degeneration with progranulin or C9ORF72 mutations

**DOI:** 10.1002/acn3.50875

**Published:** 2019-08-25

**Authors:** Nobutaka Sakae, Shanu F. Roemer, Kevin F. Bieniek, Melissa E. Murray, Matthew C. Baker, Koji Kasanuki, Neill R. Graff‐Radford, Leonard Petrucelli, Marka Van Blitterswijk, Rosa Rademakers, Dennis W. Dickson

**Affiliations:** ^1^ Department of Neuroscience Mayo Clinic Jacksonville Florida; ^2^ Department of Pathology & Laboratory Medicine University of Texas Health Science Center San Antonio Texas; ^3^ Juntendo Tokyo Koto Geriatric Medical Center Tokyo Japan; ^4^ Department of Neurology Mayo Clinic Jacksonville Florida

## Abstract

**Objective:**

To identify clinicopathological differences between frontotemporal lobar degeneration (FTLD) due to mutations in progranulin (FTLD‐GRN) and chromosome 9 open reading frame 72 (FTLD‐C9ORF72).

**Methods:**

We performed quantitative neuropathologic comparison of 17 FTLD‐C9ORF72 and 15 FTLD‐GRN with a focus on microglia. For clinical comparisons, only cases with high quality medical documentation and concurring diagnoses by at least two neurologists were included (14 FTLD‐GRN and 13 FTLD‐C9ORF72). Neuropathological analyses were limited to TDP‐43 Type A to assure consistent assessment between the groups, acknowledging that Type A is a minority of C9ORF72 patients. Furthermore, only cases with sufficient tissue from all regions were studied (11 FTLD‐GRN and 11 FTLD‐C9ORF72). FTLD cases were also compared to age– and sex–matched normal controls. Immunohistochemistry was performed for pTDP‐43, IBA‐1, CD68, and GFAP. Morphological characterization of microglia was performed in sections of cortex blinded to clinical and genetic information.

**Results:**

FTLD‐GRN patients had frequent asymmetric clinical features, including aphasia and apraxia, as well as more asymmetric cortical atrophy. Neuropathologically, FTLD‐C9ORF72 had greater hippocampal tau pathology and more TDP‐43 neuronal cytoplasmic inclusions. FTLD‐GRN had more neocortical microvacuolation, as well as more IBA‐1–positive ameboid microglia in superficial cortical layers and in subcortical white matter. FTLD‐GRN also had more microglia with nuclear condensation, possibly indicating apoptosis. Microglial morphology with CD68 immunohistochemistry in FTLD‐GRN and FTLD‐C9ORF72 differed from controls.

**Interpretation:**

Our findings underscore differences in microglial response in FTLD‐C9ORF72 and FTLD‐GRN as shown by significant differences in ameboid microglia in gray and white matter. These results suggest the differential contribution of microglial dysfunction in FTLD‐GRN and FTLD‐C9ORF72 and suggest that clinical, neuroimaging and pathologic differences could in part be related to differences in microglia response.

## Introduction

Frontotemporal lobar degeneration (FTLD) is clinically, neuropathologically and genetically heterogeneous. Among clinical presentations are changes in behavior, personality, and language. Some patients also have motor neuron disease.^1^ The most common neuropathologic findings in FTLD are tauopathies or TDP‐43 proteinopathies.^2^, ^3^ The most common genetic causes of FTLD‐TDP are mutations in progranulin (*GRN*)^4^, ^5^ and chromosome 9 open reading frame 72 (*C9ORF72*).^6^, ^7^ Mutations in *GRN* account for about one fourth^8^, ^9^ and *C9ORF72* for about one half^10^ of familial FTLD‐TDP. Clinically, almost all patients with mutations in *GRN* have frontotemporal clinical syndromes, with only rare reports of motor neuron disease.^11^ In contrast, patients with *C9ORF72* hexanucleotide repeat expansions often have motor neuron disease with or without clinical features of frontotemporal dementia.^12^

The neuropathologic features of FTLD‐TDP are focal cortical atrophy of frontal and temporal lobes with variable involvement of parietal lobe, as well as cytoplasmic inclusions in neurons and glia that are immunoreactive for TDP‐43.^13^ The relative density and distribution of neuronal and glial inclusions, as well as dystrophic neurites permits subtyping of FTLD‐TDP into Types A, B, and C, as well as less common subtypes.^14^ FTLD‐GRN is almost always Type A, while FTLD‐C9ORF72 can be Type B and less often Type A*.* 15

The pathogenesis of neurodegeneration in FTLD‐TDP remains poorly understood. Several pathomechanisms have been hypothesized for FTLD‐C9ORF72, including toxic gain of function of RNA, protein aggregation, and impairment of nucleocytoplasmic transport.^16^, ^17^ Microglial dysfunction has recently been suggested to play a role, based upon studies of *C9orf72* knock‐out mice.^18^ Mechanisms thought to play a role in FTLD‐GRN are based on the fact that progranulin has neurotrophic properties, while the proteolytic products of progranulin, the granulins, may be proinflammatory modulators.^19^ Given that FTLD‐C9ORF72 and FTLD‐GRN may have different pathogenic mechanisms, we aimed to study clinical and neuropathological differences that might be related to this fact. Several previous studies have addressed clinicopathological characteristics of FTLD‐TDP; however, few have stringently controlled for subtype. In this study, we hypothesize that different clinical features between GRN and C9orf72 could be driven by a differential role of microglia in neuroinflammation. To address this hypothesis, we measured microglial phenotypes based on density and morphology in cases of FTLD‐GRN and FTLD‐C9ORF72 with Type A TDP‐43 pathology.

## Materials and Methods

### Case materials

All cases were submitted for diagnostic studies and research to the brain bank for neurodegenerative disorders at Mayo Clinic in Jacksonville, Florida. We identified 17 cases of FTLD‐C9ORF72 with Type A TDP‐43 pathology and 15 cases of FTLD‐GRN. All cases were neuropathologically classified by a single neuropathologist (DWD), and genotyping was performed on DNA extracted from frozen brain tissue. For clinicopathological studies, cases were included only if they had good quality medical documentation and there was diagnostic concurrence by a minimum of two neurologists. The final set of cases for clinical comparisons was 13 FTLD‐C9ORF72 and 14 FTLD‐GRN. For quantitative microglial morphological studies, tissue from all brain regions had to be available for assessment. The final set of cases for pathologic studies was 11 FTLD‐C9ORF72 and 11 FTLD‐GRN. A summary of cases included in clinical and pathologic analyses are listed in Table 1 and additional details are provided in Table S1.

**Table 1:** Summary of clinical and pathologic features of cases studied.

	Demographics				Pathologic features					Clinical features							
	Case	Group	Sex	Age	Brain weight	Braak stage	Thal phase	Other	HpScl	Dur.	Memory	Behavior	EPS	Lang.	Imaging	FHx	Dx
FTLD‐C9ORF72	1	Clin	M	76	880	III	1	Fahr	−	7	+	−	−	+	L = R	+	AD
	2	Clin	M	70	880	0	0		+	10	−	+	+	+	L = R	−	bvFTD
	3	Clin	M	74	780	II–III	0		−	6	−	+	+	+	L = R	−	DLB
	4	Clin	F	83	1020	III	0	LB	+	5	+	−	−	−	L = R	−	AD
	5	Clin	M	70	1140	IV	1	LB	+	5	+	+	−	−	L > R	−	bvFTD
	6	Clin	M	80	980	V	4	CAA	+	5	+	−	−	+	L = R	−	AD
	7	Clin+Path	M	86	940	II–III	0		+	12	+	−	+	−	L = R	−	DLB
	8	Clin+Path	M	81	1000	II	1		+	7	+	−	+	+	L = R	−	AD
	9	Clin+Path	M	73	900	III–IV	0		+	3	−	+	+	+	···	+	bvFTD
	10	Clin+Path	M	71	720	II–III	2		+	6	−	+	−	+	L = R	+	bvFTD
	11	Clin+Path	M	66	1020	II	0		−	2	+	+	−	−	···	−	DLB
	12	Clin+Path	F	84	740	II–III	0	AGD	+	9	+	+	+	+	L = R	+	bvFTD
	13	Clin+Path	M	66	1120	IV	0	PART	−	6	−	+	+	−	L = R	+	bvFTD
	14	Path	M	72	1280	II	0		−	···	···	···	···	···	···	···	···
	15	Path	F	90	860	II–III	2		−	···	···	···	···	···	···	···	···
	16	Path	M	65	840	II–III	0		−	···	···	···	···	···	···	···	···
	17	Path	M	55	900	III–IV	1		−	···	···	···	···	···	···	···	···
FTLD‐GRN	18	Clin	M	56	780	0	1		+	10	+	−	−	+	R > L	+	AD
	19	Clin	F	59	900	0–I	0		−	3	−	−	−	+	L = R	+	PNFA
	20	Clin	F	85	660	0	0		+	16	+	−	−	+	L > R	+	AD
	21	Clin	M	73	940	0	1		+	15	−	−	−	+	L > R	+	PNFA
	22	Clin+Path	F	65	1100	II	1		−	2	−	−	+	−	L = R	+	aPD
	23	Clin+Path	M	75	680	I	1		+	6	−	−	−	+	L > R	+	PNFA
	24	Clin+Path	F	75	660	0	0	VaD	+	7	−	+	−	+	L > R	+	bvFTD
	25	Clin+Path	M	63	900	0	0		+	4	−	+	−	−	L = R	+	bvFTD
	26	Clin+Path	M	64	1000	0	0		+	5	−	−	+	+	L = R	+	PNFA
	27	Clin+Path	F	64	760	I	0	VaD	+	8	−	−	+	+	L = R	+	CBS
	28	Clin+Path	M	65	940	0	0	tau	+	3	−	−	+	+	L = R	+	CBS
	29	Clin+Path	F	60	1000	0	1		−	4	−	−	+	−	R > L	−	CBS
	30	Clin+Path	F	67	880	0	0		−	3	−	+	−	+	L = R	+	bvFTD
	31	Clin+Path	F	71	1080	II	3		−	2	−	+	+	−	R > L	+	bvFTD
	32	Path	M	87	660	0–I	0		+	···	···	···	···	···	···	···	···

Cases included in clinical comparisons of FTLD‐GRN and FTLD‐C9ORF72 are indicated by “Clin,” those included in pathological studies by “Path.” A subset of cases was used in both studies (“Clin + Path”). Abbreviations: M, male; F, female; Braak stage, Braak neurofibrillary tangle stage (range: 0 to VI); Thal phase, Thal amyloid phase (range: 0 to 5); HpScl, hippocampal sclerosis; Dur, disease duration; EPS, extrapyramidal signs; Lang, language impairment; FHx, family history of neurodegenerative disease; Dx, final clinical diagnosis, Fahr, globus pallidus calcification; LB, Lewy body disease; CAA, cerebral amyloid angiopathy; AGD, argyrophilic grain disease; PART, primary age‐related tauopathy; VaD, cerebrovascular disease; tau, incidental 4R tauopathy; AD, Alzheimer’s disease; bvFTD, behavioral variant of frontotemporal dementia; DLB, dementia with Lewy bodies; PNFA, progressive nonfluent aphasia; aPD, atypical Parkinsonism; CBS, corticobasal syndrome.

### Clinical assessment

Cognitive and psychiatric features were abstracted from medical records of neuropsychological and psychiatric evaluations. The clinical diagnosis of included cases fulfilled the criteria for behavioral variant frontotemporal dementia (bvFTD),^20^ progressive nonfluent aphasia (PNFA),^21^ or corticobasal syndrome (CBS).^22^ Clinical asymmetry was considered present for cases with diagnosis of PNFA and CBS or by asymmetry in motor findings from neurologic examinations.

### Genetic analyses

Frozen brain tissue was used for genotyping. All FTLD‐C9ORF72 cases had hexanucleotide repeat expansions in *C9ORF72* using a repeat–primed polymerase chain reaction method to detect expansions of the GGGGCC hexanucleotide,^6^ and for most cases, the expansions were confirmed with Southern blotting.^23^ FTLD cases with *GRN* mutations were confirmed using Sanger sequencing.^5^, ^9^

### Microscopic pathology

Samples from the middle frontal gyrus (mFCtx), superior temporal gyrus (sTCtx), inferior parietal cortex (iPCtx), primary motor cortex (MCtx), hippocampus, basal ganglia, and medulla were cut at 5 *µ*m thickness, mounted on glass slides and stained with hematoxylin and eosin (H&E). Thioflavin‐S fluorescent microscopy was performed to evaluate senile plaques and neurofibrillary tangles. A Braak neurofibrillary tangle stage and a Thal amyloid phase were derived from quantitative data, as described previously.^24^

The neuropathologic assessment included immunohistochemistry for phospho‐TDP‐43 or an antibody to a neoepitope in the midregion of TDP‐43.^25^ The TDP‐43 Type A was assigned to each case using features described in the harmonized classification.^14^ All cases had neuronal cytoplasmic inclusions, short and thin dystrophic neurites in predominantly upper cortical layers, neuronal intranuclear inclusions and perivascular astrocytic inclusions.^26^

### Immunohistochemistry

Immunohistochemical studies were performed as previously described.^27^ Histologic sections and immunostained sections were quantified with digital image analysis. Sections were immunostained using antibodies to phosphorylated TDP‐43 (pS409/410, clone 11‐9, 1:5000, Cosmobio, Tokyo, Japan), ionized calcium‐binding adaptor molecule‐1 (IBA‐1) (rabbit IG, 1:3000, Wako Chemicals, USA), CD68 (clone KP1, 1:1000, DAKO/Agilent), and glial fibrillary acidic protein (GFAP) (clone GA‐5, 1:5000, BioGenex). All immunohistochemistry was performed on a DAKO AutostainerPlus (DAKO, Santa Clara, CA) with the DAKO EnVisionTM + system‐HRP (diaminobenzidine). Normal goat serum (1:20 in TBST; Sigma, St Louis, MO, USA) was used to block non‐specific antibody binding, and 3, 3'‐diaminobenzidine (DAB) as chromogen.

## Image analysis

Digital microscopy methods have been previously described.^28^ Briefly, immunostained sections were scanned on an Aperio ScanScope XT slide scanner (Leica Biosystems Aperio, USA) producing high resolution digital images. Analyses were performed using Aperio ImageScope software in defined regions of interest (ROIs). A color deconvolution algorithm was used to count the number of pixels that had strong immunoreactivity. The output was percentage of strong positive pixels relative to the total area of the ROI. For digital analysis of the hippocampus, several ROIs were defined, including the entire hippocampus, the dentate fascia, CA4 sector, and the parahippocampal gyrus.

For microvacuolation analyses, H&E sections of mFCtx, sTCtx, and parahippocampal gyrus were evaluated. The percentage of the area occupied by microvacuolation was assessed in cortical layer II as the number of weak positive pixels divided by the total number of positive pixels according to previously published methods.^29^ Severity of microvacuolation was also assessed by semiquantitative scores (none, mild, moderate or severe), and these results were similar to those obtained by image analysis (Fig. S1).

For measurement of cortical thickness in mFCtx and sTCtx, ImageScope ruler tool was used by drawing a line perpendicular to the pial surface and the gray–white junction.

### Quantitative analysis of microglial phenotypes

**Figure 1:**
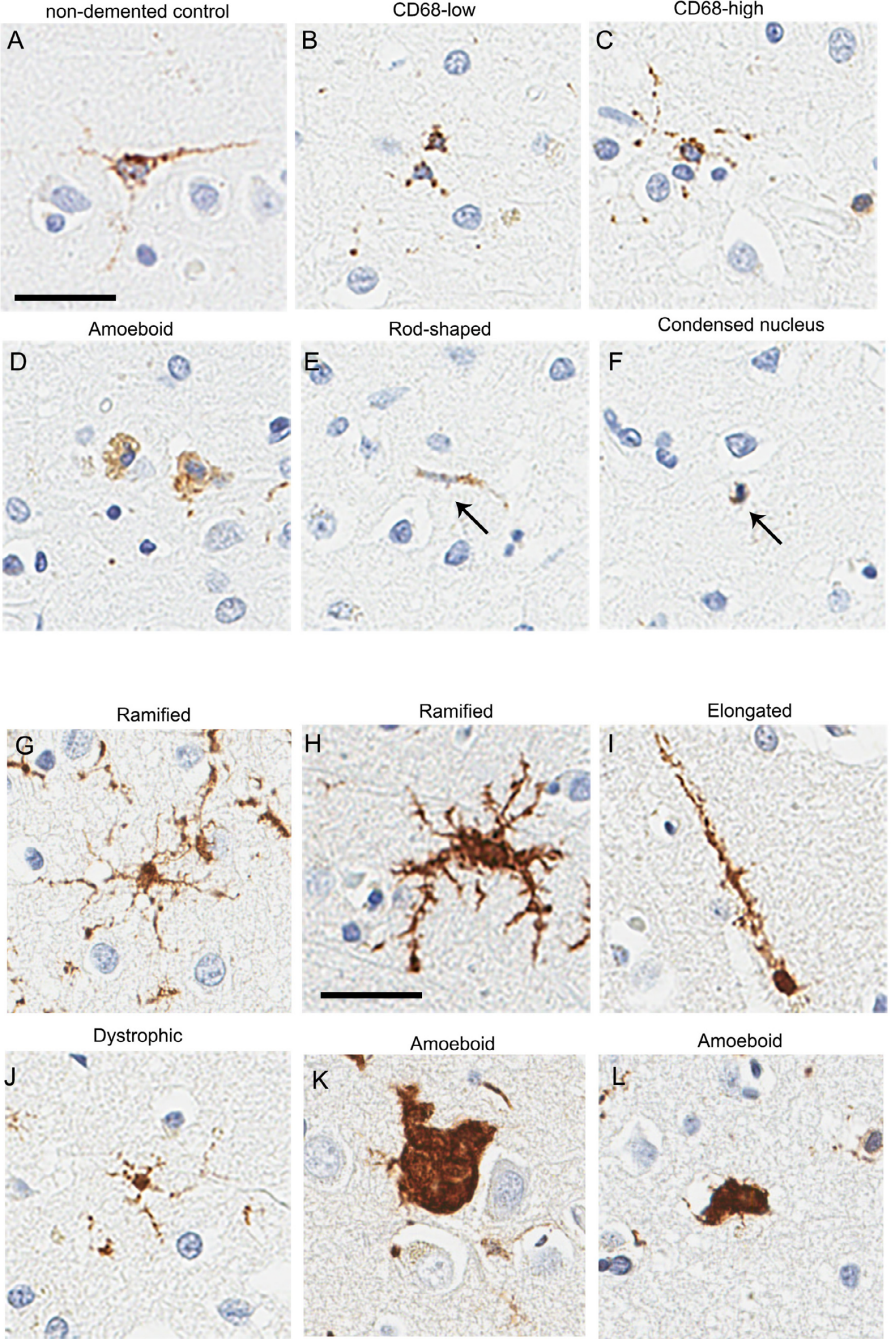
Microglial morphologic differences in FTLD‐GRN and FTLD‐C9ORF72. Examples of microglial phenotypes with CD68 immunohistochemistry: (A) typical microglia in neurologically normal control; microglial subtypes: CD68‐low (B), CD68‐high (C), ameboid (D), rod‐shaped (E), and microglia with condensed nuclei (F). Examples of microglial phenotypes with IBA‐1 immunohistochemistry: ramified microglia (G, H), elongated (I), dystrophic (J), and ameboid microglia (K, L). Scale bar = 30 *µ*m.

Digital image analysis was used to assess microglial density based on signal intensity in cell bodies and processes. To further investigate the role of microglia in FTLD‐TDP, we performed qualitative morphologic characterization of different microglia phenotypes using the Aperio counting tool for manual labeling of IBA‐1–positive or CD68–positive microglia in fixed‐sized ROI. Digital image analysis and manual counting of IBA‐stained sections gave comparable results (Fig. S2). Individual microglial subpopulations were assessed in mFCtx, a severely affected region, and compared to MCtx, a minimally affected region and in the subcortical white matter. Microglia were categorized as ramified, elongated, rod‐shaped or ameboid (Fig. 1). Dystrophic microglia had evidence of fragmentation or beading of cell processes according to the criteria of Streit and Braak.^30^ Elongated cells had a polarized appearance with minimal branching, with rod–shaped nuclei, and process lengths between 75–150 *µ*m. The number of IBA‐1–positive ameboid, ramified, elongated, and dystrophic microglia were counted in each ROI. Cells were divided into CD68‐low, which lacked immunostaining of cell processes, but had perinuclear granules (Fig. 1) and CD68‐high, which had immunostaining of cell processes. Microglial counts in defined ROIs were made by two observers, one blinded (SFR) to all clinical, genetic and pathologic information. Five neurologically normal individuals, matched for age and sex, were used as controls for this analysis (Table S2).

### Statistical analyses

SigmaPlot (Systat Software, San Jose, CA) was used for statistical analyses. Due to small sample sizes, non‐parametric Kruskal–Wallis analysis of variance (ANOVA) on ranks was performed on quantitative measures and differences in median values were assessed. Post hoc pairwise comparisons were performed between groups using Mann–Whitney rank sum test. For categorical data (sex, presence or absence of extrapyramidal signs, and symmetry/asymmetry of neuroimaging findings), a Chi‐squared test was used to compare group differences. Fisher’s exact test was used for pairwise categorical data when the count was less than 5. Correlative analysis was performed using Spearman rank order correlation. A *P*‐value of < 0.05 was considered statistically significant. Given that FTLD‐GRN tended to have more women and FTLD‐C9ORF72 to be older, analyses were also adjusted for age and sex in a multiple logistic regression models assessing clinical differences, such as memory impairment similar adjustments were made in multiple linear regression analyses for Braak stage.

## Results

### Clinical comparison of FTLD‐GRN and FTLD‐C9ORF72

#### Summary statistics of cases used in clinical analyses

A summary of demographic, genetic and clinical information on FTLD‐GRN and FTD‐C9ORF72 is shown in Table 2. The median age at death was 65 years for FTLD‐GRN and 74 years for FTLD‐C9ORF72 (*P* = 0.011). There were no statistically significant differences between the two groups with respect to median disease duration and male‐to‐female ratio; but there were more women in FTLD‐GRN (57%) than FTLD‐C9ORF72 (27%). This observation fits with a recent meta‐analyses showing that FTLD‐GRN is more common in women than men.^31^

**Table 2:** Comparison of clinical features in FTLD‐GRN and FTLD‐C9ORF72.

	FTLD‐GRN (*N* = 14)	FTLD‐C9ORF72 (*N* = 13)	*P*‐value
Demographic characteristics			
Sex (number [percent] of men)	6 (43%)	11 (81%)	0.06
Age at death	65 (62, 74)	74 (70, 82)	0.01
Disease duration	4 (3, 8)	6 (5, 8)	n.s.
Clinical features			
Behavior (apathy/disinhibition)^a^	4 (28%)	8 (62%)	n.s.
Memory disorder	2 (14%)	8 (62%)	0.02
Progressive nonfluent aphasia	4 (29%)	0 (0%)	n.s.
Progressive aphasia, not otherwise specified	3 (14%)	2 (15%)	n.s
Apraxia	3 (21%)	0 (0%)	n.s.
Limb dystonia	2 (14%)	0 (0%)	n.s.
Alien hand syndrome	1 (7%)	0 (0%)	n.s.
Clinically asymmetry	9 (63%)	2 (15%)	0.03
Limb rigidity or bradykinesia	6 (43%)	7 (54%)	n.s.
Asymmetric atrophy on neuroimaging^a^	7 (50%)	1 (8%)	0.03

Data are displayed as median (25th percentile, 75th percentile) or percent of patients with the specific feature, unless otherwise noted. Significant values (*P* < 0.05) or those trending (*P* < 0.10) are indicated. n.s., not significant.

a Percentage for patients on which information is available. Clinically asymmetry was considered as clinical diagnosis was PNFA, CBS, laterality of extrapyramidal sign.

#### Asymmetric clinical syndromes and neuroimaging findings

The asymmetrical clinical syndrome of CBS was noted in three of 14 patients (21%) with FTLD‐GRN, but in none of the FTLD‐C9ORF72 patients. Available MRI or CT images (14 for FTLD‐GRN and 11 for FTLD‐C9ORF72) showed asymmetrical cortical atrophy in only one patient with FTLD‐C9ORF72, but in seven (50%) patients with FTLD‐GRN (Table 2).

### Language disorders

Progressive aphasia (four PNFA and two progressive aphasia not otherwise specified) was noted in six of 14 patients with FTLD‐GRN, but in only three of 13 FTLD‐C9ORF72 patients. One patient appeared to have logopenic aphasia, and one patient appeared to have semantic components. A receptive component could not be excluded confidently. The remainder clearly had progressive aphasia, but could not be further subtyped.

#### Amnestic clinical syndromes and extrapyramidal signs

Amnestic dementia was based on neuropsychological evaluations and considered for patients where memory complaints were the predominant feature. Alzhiemer dementia was in the clinical differential diagnosis of some of these patients. Memory problems overshadowed other clinical features of FTLD, such as behavioral and language problems. Amnestic dementia (AD) was prominent in two patients (14%) with FTLD‐GRN, but in eight patients (62%) with FTLD‐C9ORF72. AD was the clinical diagnosis of four of eight FTLD‐C9ORF72 patients (Table 1). In FTLD‐C9ORF72, prominent memory problems were noted in four patients with dementia with Lewy bodies (DLB) (31%), and these patients also had extrapyramidal signs and visual hallucinations. The frequency of extrapyramidal signs in the entire cohort was similar in FTLD‐GRN and FTLD‐C9ORF72 (Table 2).

#### Effects of age and Alzheimer type pathology on clinical features

To determine potential confounding variables accounting for observed differences in clinical syndromes and neuroimaging findings, we addressed possible contributing pathologic features, such as brain weight, Thal amyloid phase, Braak neurofibrillary tangle (NFT) stage, and presence of hippocampal sclerosis (HpScl). There were no differences between FTLD‐GRN and FTLD‐C9ORF72 for age at death, disease duration, brain weight, frequency of HpScl or Thal amyloid phase. On the other hand, the median Braak NFT stage was greater (*P* < 0.001) in FTLD‐C9ORF72 than in FTLD‐GRN. To adjust for the effects of age at death and sex on differences in Braak NFT stage, a multiple linear regression model showed higher (*P* = 0.011) Braak NFT stage in FTLD‐C9ORF72 compared with FTLD‐GRN.

#### TDP‐43 pathology

**Figure 2:**
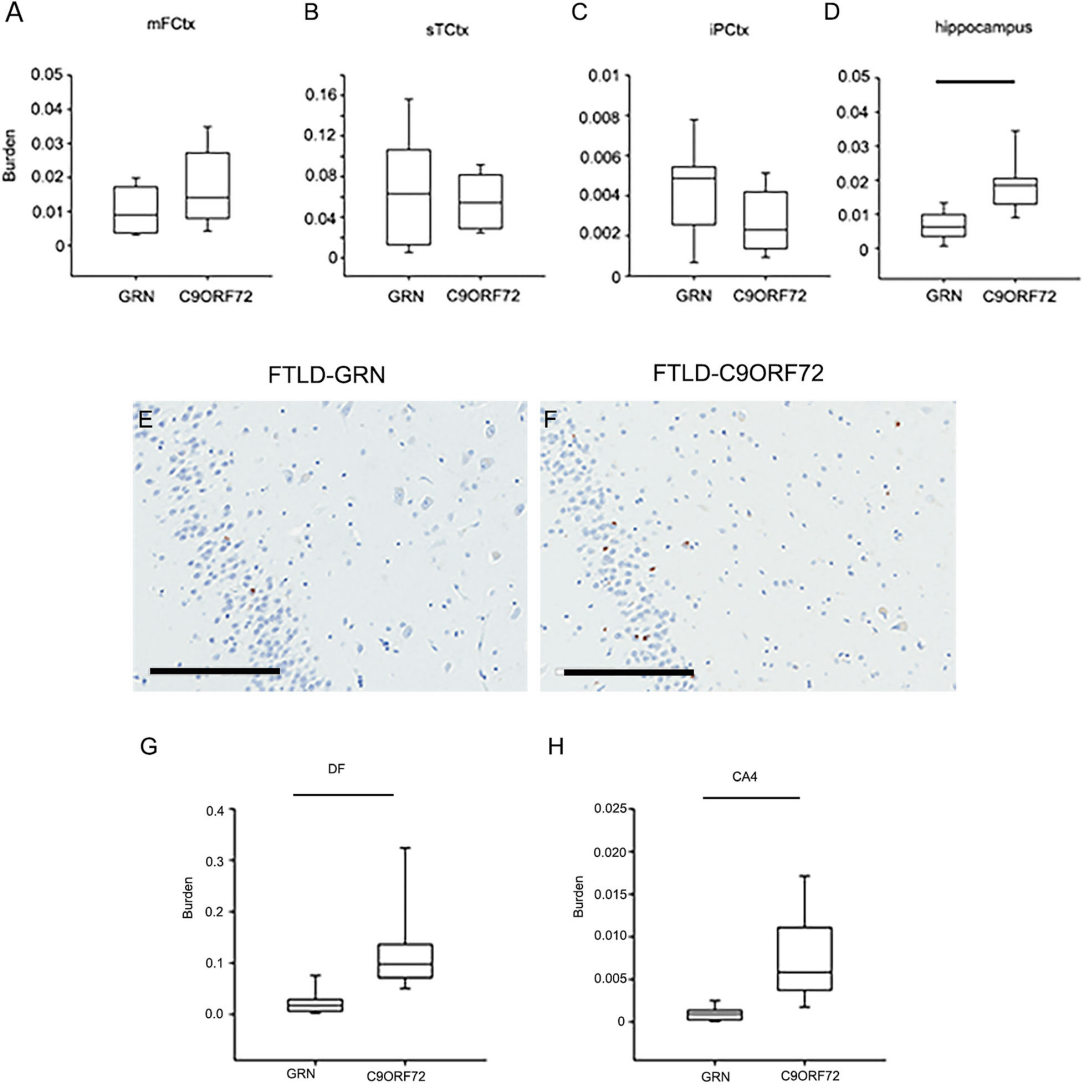
TDP‐43 burden in FTLD‐GRN and FTLD‐C9ORF72. TDP‐43 burden was calculated as the proportion of the total area with positive signal using pTDP‐43 immunohistochemistry in mFCtx (A), sTCtx (B), iPCtx (C), and hippocampus (D). Representative images of TDP‐43 pathology in dentate fascia and endplate in FTLD‐GRN (E) and FTLD‐C9ORF72 (F). Note the sparse neuronal cytoplasmic inclusions in FTLD‐GRN and more numerous inclusions in FTLD‐C9ORF72. Quantitative analysis shows significantly greater TDP‐43 burden in FTLD‐C9ORF72 compared with FTLD‐GRN in both dentate fascia (DF) (G) and endplate (CA4) (H). Box plots show median (line) and 25th and 75th percentiles; whiskers show 10th and 90th percentiles. Scale bars in (B) = 200 *µ*m. MFCtx, middle frontal gyrus; sTCtx, superior temporal gyrus; iPCtx, inferior parietal cortex; hippocampus, entire area of hippocampus. Bars indicate *P* < 0.05 with Mann–Whitney *U* test.

To assess differences in TDP‐43 pathology histological sections from the mFCtx, sTCtx, iPCtx and hippocampus were assessed and scored semiquantitatively as either present or absent. The density of phospho‐TDP‐43 pathology was also assessed with image analysis. There were no significant differences in TDP‐43 pathology in MCtx (not shown); however, densities of TDP‐43 pathology tended to be greater in FTLD‐GRN than FTLD‐C9ORF72 in mFCtx, sTCtx and iPCtx, (Fig. 2). On the other hand, FTLD‐C9ORF72 had greater TDP‐43 density in the hippocampus than FTLD‐GRN (*P* = 0.002). In subregions of the hippocampus, TDP‐43 density was significantly greater in dentate gyrus (*P* < 0.001) and CA4 sector (*P* < 0.001) in FTLD‐C9ORF72 compared with FTLD‐GRN.

#### Cortical thickness

We assessed cortical thickness in mFCtx and sTCtx. FTLD‐GRN had significant atrophy of the mFCtx (*P* = 0.036) (Table 3). The cortical thickness in the sTCtx was not significantly different.

**Table 3:** Cortical thickness and CD68–positive microglia FTLD‐GRN and FTLD‐C9ORF72.

	FTLD‐GRN	FTLD‐C9ORF72	Ctrl	*P*‐value
Cortical thickness (mFCtx)	1845 (1605, 1959)^2^	2070 (1800, 2460)	2695 (2217, 2697)	0.036
Comparison of CD68–positive microglia (mm^2^)				
CD‐68 low	24 (16, 41)	24 (14, 35)	18 (8, 20)	
CD68‐high	5 (2, 16)	8 (4, 21)	59 (50, 68)^1^	0.002
Ameboid	1 (0, 6)	1 (0, 1)	1 (1, 3)	
Rod‐shaped	7 (2, 12)^2^	2 (1, 4)	1 (0, 1)	0.02
Condensed nucleus	9 (4, 14)^3^	1 (0, 1)	1 (0, 1)	<0.001
Comparison of IBA‐1–positive microglia (mm^2^)				
Ramified	50 (25, 175)	75 (25, 125)	52 (52, 67)	
Ameboid	3 (3, 7)^2^	1 (0, 3)	1 (0, 1)	0.01
Rod‐shaped	50 (13, 150)^2^	13 (0, 50)	0 (0, 1)	0.009
Dystrophic	188 (113, 250)	250 (200, 312)	0 (0, 0)^1^	0.001

Cortical thickness measured in middle frontal gyrus (mFCtx) (expressed in mm (millimeters)). Manual counts of CD68‐positive microglia and IBA‐1‐positive microglia in mFCtx in FTLD‐GRN and FTLD‐C9ORF72 with respect to different morphologic subtypes. All data are displayed as median (25th percentile, 75th percentile). *P*‐value for ANOVA on Ranks comparison of all three groups.

^1^ Ctrl versus FTLD.

^2^ GRN versus Ctrl.

^3^ GRN versus Ctrl and C9.

#### Neocortical microvacuolation

**Figure 3:**
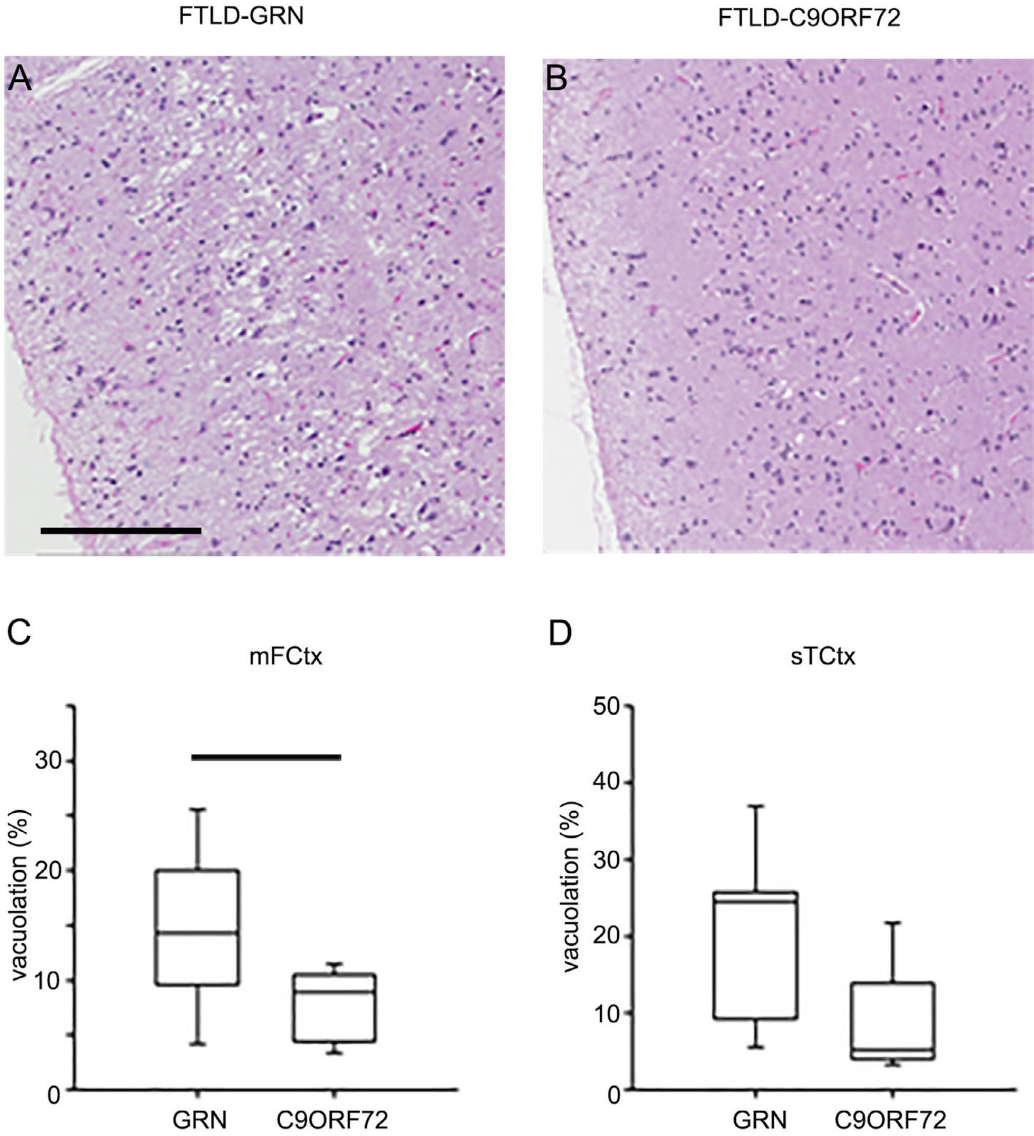
Cortical atrophy and superficial spongiosis in FTLD‐GRN and FTLD‐C9ORF72. Representative images of microvacuolation (MV) in superficial cortical layers of FTLD‐GRN (A) and FTLD‐C9ORF72 (B). Image analysis of the percent area of superficial cortex occupied by vacuoles (%). Vacuolation is significantly greater in FTLD‐GRN compared with FTLD‐C9ORF72 in middle frontal gyrus (mFCtx) (C), with a similar, but not statistically significant trend in the superior temporal gyrus (sTCtx) (D). Box plots show median (line) and 25th and 75th percentiles; whiskers show 10th and 90th percentiles. Bar indicates *P* < 0.05 with Mann–Whitney *U* test. (Scale bar = 150 *µ*m).

The degree of superficial microvacuolation was assessed using digital image analysis in the mFCtx and sTCtx. In most cases, at least some degree of microvacuolation was detected in both ROIs (Fig. 3). Quantitative analysis revealed that microvacuolation was more severe in FTLD‐GRN than FTLD‐C9ORF72, reaching statistical significance in mFCtx (*P* = 0.036).

#### Neuroinflammation

There were no significant differences in overall density of IBA‐1, CD68 and GFAP‐positive glial cells between FTLD‐C9ORF72 and FTLD‐GRN using digital image analysis in mFCTx, sTCTx, iPCTx, MCTx. The only exception was more CD68–positive microglia in the hippocampus in FTLD‐C9ORF72 (data not shown).

#### Manual morphological assessment of IBA‐1– and CD68–positive microglia

**Figure 4:**
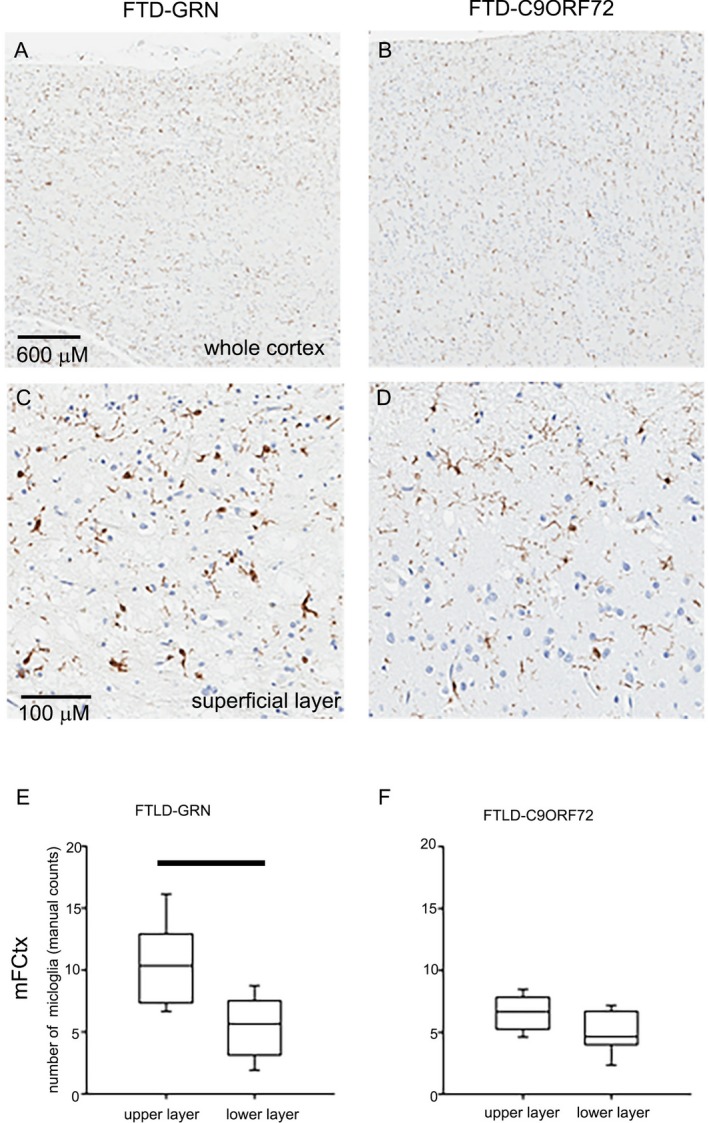
Cortical IBA‐1–positive cells in FTLD‐GRN and FTLD‐C9ORF72. Representative images show entire cortical thickness of middle frontal gyrus (A and B) and superficial cortical layers (C and D) with IBA‐1 immunohistochemistry. Box plots (E and F) show the density of IBA‐1–positive cells in the upper and lower cortical layers by manual counts, relative to the entire region of interest. Box plots show median (line) and 25th and 75th percentiles; whiskers show 10th and 90th percentiles. Bars indicate *P* < 0.05 with Mann–Whitney *U* test.

**Figure 5:**
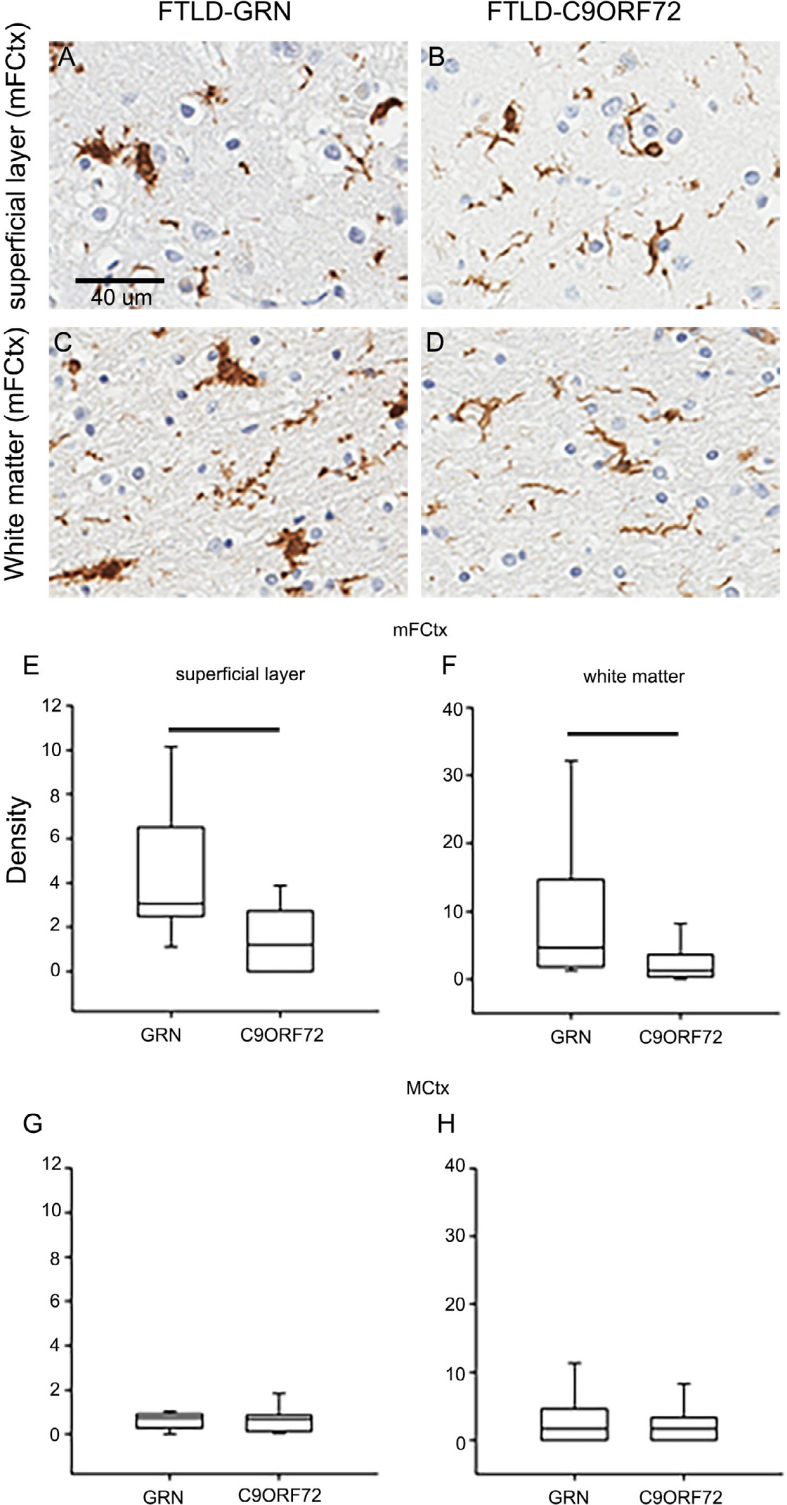
IBA‐1–positive ameboid cells in FTLD‐GRN and FTLD‐C9ORF72. Comparison of ameboid microglia in superficial cortical layer (A and B) and subcortical white matter (C and D) of middle frontal gyrus (mFCtx) in FTLD‐GRN and FTLD‐C9ORF72. (Scale bar = 40 *µ*m). Density is the number of IBA‐1–positive ameboid microglia in superficial cortical layers (E) and subcortical white matter (F). Similar assessments in motor cortex (MCtx) show lower density of microglia in both superficial cortical layers (G) and subcortical white matter (H). Box plots show median (line) and 25th and 75th percentiles; whiskers show 10th and 90th percentiles. Bars indicate *P* < 0.05 with Mann–Whitney *U* test.

Both FTLD‐GRN and FTLD‐C9ORF72 showed significantly fewer CD68‐high microglia than normal controls. FTLD‐GRN had significantly more rod–shaped microglia, and more microglia with condensed nuclei compared with both controls and FTLD‐C9ORF72 (*P* < 0.001). FTLD‐GRN also had more IBA‐1‐positive and CD68‐positive rod‐shaped microglia than controls (*P* = 0.002). Compared to normal controls, both FTLD‐GRN and FTLD‐C9ORF72 had significantly more dystrophic microglia in mFCtx (Table 3). Interestingly, the number of total IBA‐1–positive cells in mFCtx was significantly greater in upper than lower cortical layers in FTLD‐GRN. In contrast, an minimally affected cortical region (MCtx), did not show any significant differences between FTLD‐GRN and FTLD‐C9ORF72 (Fig. 4). Superficial cortical layers in mFCtx not only had greater microvacuolation in FTLD‐GRN, but also significantly greater density of ameboid microglia compared with FTLD‐C9ORF72 (*P* = 0.025). Similarly, more ameboid microglia were detected in the deep white matter of mFCtx in FTLD‐GRN compared with FTLD‐C9ORF72 (*P* = 0.038). MCtx showed no significant difference between FTLD‐GRN and FTLD‐C9ORF72 (Fig. 5).

## Discussion

In this study, we compared FTLD‐GRN and FTLD‐C9ORF72 matched for TDP‐43 subtype (Type A) to exclude for differences due to TDP‐43 subtype. Although there are several clinical, neuropsychological, and radiological studies of FTLD‐GRN and C9ORF72, to our knowledge, this is the first direct clinicopathological comparison of FTLD‐GRN and FTLD‐C9ORF72 matched for TDP‐43 type.

In our clinical comparison, we found that FTLD‐C9ORF72 more frequently had amnestic dementia, including antemortem diagnoses of AD or DLB, as well as more symmetrical and milder cortical atrophy compared with FTLD‐GRN. Although amnestic deficits in FTD can be affected by executive dysfunction,^32^ our observations are in line with previous reports, including Mahoney and coworkers and Simon‐Sanchez and coworkers, who reported about half of FTLD‐C9ORF72 patients presented with memory impairment.^33^, ^34^ Our previous study showed FTLD‐C9ORF72 had greater tau pathology than FTLD‐GRN. A previous study showed less tau pathology in FTLD‐GRN compared to FTLD‐C9ORF72.^27^ In this study, we similarly found decreased tau densities in FTLD‐GRN in the mFCtx and hippocampus compared to FTLD‐C9ORF72. In line with this, Papegaey and coworkers biochemically reported reduction of tau protein expression in frontal cortex of FTLD‐GRN.^35^ We also found significant greater TDP‐43 in the hippocampus of FTLD‐C9ORF72 compared with FTLD‐GRN in addition to tau pathology. It is likely that hippocampal TDP‐43 pathology and tau pathology both contribute to the amnestic phenotype in FTLD‐C9ORF72.

FTLD‐GRN patients more often had asymmetrical clinical presentations, such as PNFA and CBS. This is in line with previous reports,^36^, ^37^ including a study by Pickering‐Brown and coworkers who reported that PNFA was more common in FTLD‐GRN (36%).^38^ FTLD‐GRN also had more severe cortical atrophy, microvacuolation, and more ameboid microglia in cortical regions (but not in the hippocampus) compared with FTLD‐C9ORF72. More severe and asymmetrical cortical atrophy, as well as greater TDP‐43 pathology in, sTCtx and iPCtx may underlie a pathological substrate of aphasia.

In the brain, expression studies based upon RNA sequencing show that it is extremely high in microglia and sparse in neurons.^39^ Loss of function mutations in *GRN*, leading to nonsense mediated decay of mRNA,^9^ are thought to be associated with microglial dysfunction.^40^, ^41^

There are only a few quantitative neuropathologic studies on neuroinflammation in FTLD‐GRN and FTLD‐C9ORF72. Lant and coworkers performed a semiquantitative analysis of CD68–positive microglia in FTLD‐GRN (Type A), FTLD‐C9ORF72 (Types A and B) and FTLD‐MAPT in frontal and temporal cortices and found significantly more CD68–positive microglia in FTLD‐MAPT than in FTLD‐GRN and FTLD‐C9ORF72, but no difference between the two genetic forms of FTLD‐TDP.^42^

In the present study, however, we assessed critically affected areas such as cortical layer II and also subtyped microglia based on morphology. We did not find any significant differences in rod–shaped or dystrophic microglia between FTLD‐GRN and FTLD‐C9ORF72, but showed that FTLD had significantly more dystrophic microglia compared to control. Several studies highlight a role for rod–shaped and dystrophic microglia in neurodegenerative diseases.^30^, ^43^, ^44^ The exact significance of these findings remain to be identified, but may be related to microglial dysfunction. On the other hand, FTLD‐GRN had more microglia with condensed nuclei, which may suggest either increased vulnerability of microglia deficient in GRN or an increased turnover of macrophages.

Furthermore, FTLD‐GRN had significantly more IBA‐1–positive ameboid cells in layer II of mFCtx and deep white matter. This is consistent with a study by Woollacott and coworkers reporting high density of amoeboid microglia in the cortex and white matter of a single FTLD‐GRN case.^45^ These findings suggest a role for ameboid microglia in the pathogenesis of both white matter and gray matter damage in FTLD‐GRN. FTLD‐GRN also showed more significant neocortical microvacuolation. The underlying molecular mechanism for microvacuolation is unknown, but synaptic damage has long been presumed to play a role.^46^ Lui and coworkers showed that progranulin knock‐out mice have synaptic loss through defective synaptic pruning by microglia.^40^ They also found increased IBA‐1–positive microglia in the frontal cortex of FTLD‐GRN compared with neurological controls.^40^ In conclusion, we found distinct clinicopathological differences between in FTLD‐GRN and FTLD‐C9ORF72 with respect to type A TDP‐43. Clinical asymmetric phenotypes, such as PNFA or CBS, as well as asymmetric brain atrophy, were more common in FTLD‐GRN. Early memory deficits and symmetric brain atrophy were more common in FTLD‐C9ORF72. Our neuropathological analyses highlight differential neocortical microvacuolation and phagocytic microglial phenotype between FTLD‐GRN and FTLD‐C9ORF72. Microglial dysfunction may be implicated in both mutations, but our data suggest different roles for neuroinflammation between FTLD‐GRN and FTLD‐C9ORF72. Additional experimental studies are warranted to better determine shared or distinct downstream mechanisms in microglial function associated with mutations in *C9orf72* and *GRN*. Furthermore, future clinicopathological studies should include comparative studies not only of FTLD Type A, which is found in a minority of C9ORF72 patients, and thus may not be representative of most C9ORF72 patients.

## Author Contributions

N.S. and D.W.D. provided conception and design of the study. N.S., S.F.R., K.K., M.B., R.R., M.B., provided acquisition and analysis of data. N.S. and S.F.R drafted the manuscript and figures. R.N, L.P, and D.W.D. provided funding. K.F.B., S.F.R, K.K., M.B., N.G.R. and D.W.D. provided critical revisions of the manuscript.

## Conflict of Interest

None of the authors has any real or perceived conflicts of interest.

## Supporting information


**Table S1.** Demographic and clinical features of FTLD cases.
**Table S2.** Demographic and pathologic features of FTLD cases and normal controls used in microglial morphologic studies.

**Figure S1.** Comparison of manual microglial counts to image analysis of IBA‐1 density.

**Figure S2.** Comparison of manual microvacuolation scores and vacuolation burden from image analysis.

## References

[1] Rademakers R , Neumann M , Mackenzie IR . Advances in understanding the molecular basis of frontotemporal dementia. Nat Rev Neurol 2012;8:423–434. 22732773 10.1038/nrneurol.2012.117 PMC3629543

[2] Irwin DJ , Cairns NJ , Grossman M , et al. Frontotemporal lobar degeneration: defining phenotypic diversity through personalized medicine. Acta Neuropathol 2015;129:469–491. 25549971 10.1007/s00401-014-1380-1 PMC4369168

[3] Goossens J , Vanmechelen E , Trojanowski JQ , et al. TDP‐43 as a possible biomarker for frontotemporal lobar degeneration: a systematic review of existing antibodies. Acta Neuropathol Commun 2015;3:15. 25853864 10.1186/s40478-015-0195-1 PMC4380254

[4] Cruts M , Gijselinck I , van der Zee J , et al. Null mutations in progranulin cause ubiquitin‐positive frontotemporal dementia linked to chromosome 17q21. Nature 2006;442:920–924. 16862115 10.1038/nature05017

[5] Baker M , Mackenzie IR , Pickering‐Brown SM , et al. Mutations in progranulin cause tau‐negative frontotemporal dementia linked to chromosome 17. Nature 2006;442:916–919. 16862116 10.1038/nature05016

[6] DeJesus‐Hernandez M , Mackenzie IR , Boeve BF , et al. Expanded GGGGCC hexanucleotide repeat in noncoding region of C9ORF72 causes chromosome 9p‐linked FTD and ALS. Neuron 2011;72:245–256. 21944778 10.1016/j.neuron.2011.09.011 PMC3202986

[7] Renton AE , Majounie E , Waite A , et al. A hexanucleotide repeat expansion in C9ORF72 is the cause of chromosome 9p21‐linked ALS‐FTD. Neuron 2011;72:257–268. 21944779 10.1016/j.neuron.2011.09.010 PMC3200438

[8] Petkau TL , Leavitt BR . Progranulin in neurodegenerative disease. Trends Neurosci 2014;37:388–398. 24800652 10.1016/j.tins.2014.04.003

[9] Gass J , Cannon A , Mackenzie IR , et al. Mutations in progranulin are a major cause of ubiquitin‐positive frontotemporal lobar degeneration. Hum Mol Genet 2006;15:2988–3001. 16950801 10.1093/hmg/ddl241

[10] Sieben A , Van Langenhove T , Engelborghs S , et al. The genetics and neuropathology of frontotemporal lobar degeneration. Acta Neuropathol 2012;124:353–372. 22890575 10.1007/s00401-012-1029-x PMC3422616

[11] Cannon A , Fujioka S , Rutherford NJ , et al. Clinicopathologic variability of the GRN A9D mutation, including amyotrophic lateral sclerosis. Neurology 2013;80:1771–1777. 23596077 10.1212/WNL.0b013e3182919059 PMC3719429

[12] Boeve BF , Boylan KB , Graff‐Radford NR , et al. Characterization of frontotemporal dementia and/or amyotrophic lateral sclerosis associated with the GGGGCC repeat expansion in C9ORF72. Brain 2012;135:765–783. 22366793 10.1093/brain/aws004 PMC3286335

[13] Cairns NJ , Bigio EH , Mackenzie IR , et al. Neuropathologic diagnostic and nosologic criteria for frontotemporal lobar degeneration: consensus of the Consortium for Frontotemporal Lobar Degeneration. Acta Neuropathol 2007;114:5–22. 17579875 10.1007/s00401-007-0237-2 PMC2827877

[14] Mackenzie IR , Neumann M , Baborie A , et al. A harmonized classification system for FTLD‐TDP pathology. Acta Neuropathol 2011;122:111–113. 21644037 10.1007/s00401-011-0845-8 PMC3285143

[15] Mackenzie IR , Frick P , Grasser FA , et al. Quantitative analysis and clinico‐pathological correlations of different dipeptide repeat protein pathologies in C9ORF72 mutation carriers. Acta Neuropathol 2015;130:845–861. 26374446 10.1007/s00401-015-1476-2

[16] Stepto A , Gallo JM , Shaw CE , Hirth F . Modelling C9ORF72 hexanucleotide repeat expansion in amyotrophic lateral sclerosis and frontotemporal dementia. Acta Neuropathol 2014;127:377–389. 24366528 10.1007/s00401-013-1235-1

[17] Cruts M , Gijselinck I , Van Langenhove T , et al. Current insights into the C9orf72 repeat expansion diseases of the FTLD/ALS spectrum. Trends Neurosci 2013;36:450–459. 23746459 10.1016/j.tins.2013.04.010

[18] O'Rourke JG , Bogdanik L , Yanez A , et al. C9orf72 is required for proper macrophage and microglial function in mice. Science 2016;351:1324–1329. 26989253 10.1126/science.aaf1064 PMC5120541

[19] Holler CJ , Taylor G , Deng Q , Kukar T . Intracellular proteolysis of progranulin generates stable, lysosomal granulins that are haploinsufficient in patients with frontotemporal dementia caused by GRN mutations. eNeuro 2017;4 10.1523/ENEURO.0100-17.2017 PMC5562298 28828399

[20] Neary D , Snowden JS , Gustafson L , et al. Frontotemporal lobar degeneration: a consensus on clinical diagnostic criteria. Neurology 1998;51:1546–1554. 9855500 10.1212/wnl.51.6.1546

[21] Gorno‐Tempini ML , Hillis AE , Weintraub S , et al. Classification of primary progressive aphasia and its variants. Neurology 2011;76:1006–1014. 21325651 10.1212/WNL.0b013e31821103e6 PMC3059138

[22] Armstrong MJ , Litvan I , Lang AE , et al. Criteria for the diagnosis of corticobasal degeneration. Neurology 2013;80:496–503. 23359374 10.1212/WNL.0b013e31827f0fd1 PMC3590050

[23] van Blitterswijk M , DeJesus‐Hernandez M , Niemantsverdriet E , et al. Association between repeat sizes and clinical and pathological characteristics in carriers of C9ORF72 repeat expansions (Xpansize‐72): a cross‐sectional cohort study. Lancet Neurol 2013;12:978–988. 24011653 10.1016/S1474-4422(13)70210-2 PMC3879782

[24] Murray ME , Lowe VJ , Graff‐Radford NR , et al. Clinicopathologic and 11C‐Pittsburgh compound B implications of Thal amyloid phase across the Alzheimer's disease spectrum. Brain 2015;138:1370–1381. 25805643 10.1093/brain/awv050 PMC4407190

[25] Zhang YJ , Xu YF , Cook C , et al. Aberrant cleavage of TDP‐43 enhances aggregation and cellular toxicity. Proc Natl Acad Sci USA 2009;106:7607–7612. 19383787 10.1073/pnas.0900688106 PMC2671323

[26] Lin WL , Castanedes‐Casey M , Dickson DW . Transactivation response DNA‐binding protein 43 microvasculopathy in frontotemporal degeneration and familial Lewy body disease. J Neuropathol Exp Neurol 2009;68:1167–1176. 19816201 10.1097/NEN.0b013e3181baacec PMC2783428

[27] Bieniek KF , Murray ME , Rutherford NJ , et al. Tau pathology in frontotemporal lobar degeneration with C9ORF72 hexanucleotide repeat expansion. Acta Neuropathol 2013;125:289–302. 23053135 10.1007/s00401-012-1048-7 PMC3551994

[28] Sakae N , Bieniek KF , Zhang YJ , et al. Poly‐GR dipeptide repeat polymers correlate with neurodegeneration and Clinicopathological subtypes in C9ORF72‐related brain disease. Acta Neuropathol Commun 2018;6:63. 30029693 10.1186/s40478-018-0564-7 PMC6054740

[29] Murray ME , Vemuri P , Preboske GM , et al. A quantitative postmortem MRI design sensitive to white matter hyperintensity differences and their relationship with underlying pathology. J Neuropathol Exp Neurol 2012;71:1113–1122. 23147507 10.1097/NEN.0b013e318277387e PMC3511604

[30] Streit WJ , Braak H , Xue QS , Bechmann I . Dystrophic (senescent) rather than activated microglial cells are associated with tau pathology and likely precede neurodegeneration in Alzheimer's disease. Acta Neuropathol 2009;118:475–485. 19513731 10.1007/s00401-009-0556-6 PMC2737117

[31] Curtis AF , Masellis M , Hsiung GR , et al. Sex differences in the prevalence of genetic mutations in FTD and ALS: a meta‐analysis. Neurology 2017;89:1633–1642. 28916533 10.1212/WNL.0000000000004494 PMC5634668

[32] Hornberger M , Piguet O . Episodic memory in frontotemporal dementia: a critical review. Brain 2012;135:678–692. 22366790 10.1093/brain/aws011

[33] Mahoney CJ , Beck J , Rohrer JD , et al. Frontotemporal dementia with the C9ORF72 hexanucleotide repeat expansion: clinical, neuroanatomical and neuropathological features. Brain 2012;135:736–750. 22366791 10.1093/brain/awr361 PMC3286330

[34] Simon‐Sanchez J , Dopper EG , Cohn‐Hokke PE , et al. The clinical and pathological phenotype of C9ORF72 hexanucleotide repeat expansions. Brain 2012;135:723–735. 22300876 10.1093/brain/awr353

[35] Papegaey A , Eddarkaoui S , Deramecourt V , et al. Reduced Tau protein expression is associated with frontotemporal degeneration with progranulin mutation. Acta Neuropathol Commun 2016;4:74. 27435172 10.1186/s40478-016-0345-0 PMC4952067

[36] Le Ber I , Camuzat A , Hannequin D , et al. Phenotype variability in progranulin mutation carriers: a clinical, neuropsychological, imaging and genetic study. Brain 2008;131:732–746. 18245784 10.1093/brain/awn012

[37] Beck J , Rohrer JD , Campbell T , et al. A distinct clinical, neuropsychological and radiological phenotype is associated with progranulin gene mutations in a large UK series. Brain 2008;131:706–720. 18234697 10.1093/brain/awm320 PMC2577762

[38] Pickering‐Brown SM , Rollinson S , Du Plessis D , et al. Frequency and clinical characteristics of progranulin mutation carriers in the Manchester frontotemporal lobar degeneration cohort: comparison with patients with MAPT and no known mutations. Brain 2008;131:721–731. 18192287 10.1093/brain/awm331

[39] Zhang Y , Chen K , Sloan SA , et al. An RNA‐sequencing transcriptome and splicing database of glia, neurons, and vascular cells of the cerebral cortex. J Neurosci 2014;34:11929–11947. 25186741 10.1523/JNEUROSCI.1860-14.2014 PMC4152602

[40] Lui H , Zhang J , Makinson SR , et al. Progranulin deficiency promotes circuit‐specific synaptic pruning by microglia via complement activation. Cell 2016;165:921–935. 27114033 10.1016/j.cell.2016.04.001 PMC4860138

[41] Minami SS , Min SW , Krabbe G , et al. Progranulin protects against amyloid beta deposition and toxicity in Alzheimer's disease mouse models. Nat Med 2014;20:1157–1164. 25261995 10.1038/nm.3672 PMC4196723

[42] Lant SB , Robinson AC , Thompson JC , et al. Patterns of microglial cell activation in frontotemporal lobar degeneration. Neuropathol Appl Neurobiol 2014;40:686–696. 24117616 10.1111/nan.12092

[43] Lewandowska E , Wierzba‐Bobrowicz T , Kosno‐Kruszewska E , et al. Ultrastructural evaluation of activated forms of microglia in human brain in selected neurological diseases (SSPE, Wilson's disease and Alzheimer's disease). Folia Neuropathol 2004;42:81–91. 15266782

[44] Bachstetter AD , Van Eldik LJ , Schmitt FA , et al. Disease‐related microglia heterogeneity in the hippocampus of Alzheimer's disease, dementia with Lewy bodies, and hippocampal sclerosis of aging. Acta Neuropathol Commun 2015;3:32. 26001591 10.1186/s40478-015-0209-z PMC4489160

[45] Woollacott IOC , Bocchetta M , Sudre CH , et al. Pathological correlates of white matter hyperintensities in a case of progranulin mutation associated frontotemporal dementia. Neurocase 2018;24:166–174. 30112957 10.1080/13554794.2018.1506039 PMC6168954

[46] Brun A , Liu X , Erikson C . Synapse loss and gliosis in the molecular layer of the cerebral cortex in Alzheimer's disease and in frontal lobe degeneration. Neurodegeneration 1995;4:171–177. 7583681 10.1006/neur.1995.0021

