# Supplementary material for: Microglia in frontotemporal lobar degeneration with progranulin or C9ORF72 mutations
Source: Ann Clin Transl Neurol. 2019 Aug 25;6(9):1782–96. doi: 10.1002/acn3.50875 (PMC6764493; doi:10.1002/acn3.50875)
Supplement: Supplementary file 1 — Table S1. Demographic and clinical features of FTLD cases. Table S2. Demographic and pathologic features of FTLD cases and normal controls used in microglial morphologic studies. [file ACN3-6-1782-s003.docx]

**Supplementary Table 1. Demographic and clinical features of FTLD cases**

|  | **FTLD-GRN**  (N=14) | **FTLD-C9ORF72**  (N=13) | **p-value** |
| --- | --- | --- | --- |
| **Demographic characteristics** | |  |  |
| Male | 6 (43%) | 11 (81%) | n.s. |
| Age at death (years) | 65 (62.5, 74) | 74 (70, 82) | 0.01 |
| Disease duration (years) | 4.5 (3, 8.5) | 6 (5, 8) | n.s. |
| **Pathological features** | |  |  |
| Brain weight (grams) | 900 (740, 1000) | 940 (830, 1020) | n.s. |
| Braak NFT stage | 0 (0, I) | 2 (2, 4) | <0.001 |
| Thal amyloid phase | 0 (0, I) | 0 (0, 1) | n.s. |

**Supplementary Table 2. Summary of demographic and pathologic features of FTLD cases and controls used in microglial morphologic studies**

|  | **FTLD-GRN** | **FTLD-C9ORF72** | **Normal controls** | **P-value** |
| --- | --- | --- | --- | --- |
| **Demographic characteristics** | |  |  |  |
| Number | 11 | 11 | 5 |  |
| Male | 5 (45%) | 9 (81%) | 4 (80%) | n.s. |
| Age at death (years) | 65 (64, 75) | 72 (66, 84) | 71 (66, 78) | n.s. |
| Disease duration (years) | 4 (3, 7) | 6 (2.5, 8) | NA | n.s. |
| **Pathological features** |  |  |  |  |
| Brain weight (grams) | 900 (840, 1020) | 940 (760, 1080) | 1170 (1065, 1325) | n.s. |
| Braak NFT stage | 0 (0, 1) | 2.5 (2, 3.5)* | 0.5 (0.25, 3) | <0.001 |
| Thal amyloid phase | 0 (0,1) | 0 (0, 0.75) | 0 (0, 1.5) | n.s. |
