# Supplementary figures and images for: Microglia in frontotemporal lobar degeneration with progranulin or C9ORF72 mutations
Source: Ann Clin Transl Neurol. 2019 Aug 25;6(9):1782–96. doi: 10.1002/acn3.50875 (PMC6764493; doi:10.1002/acn3.50875)

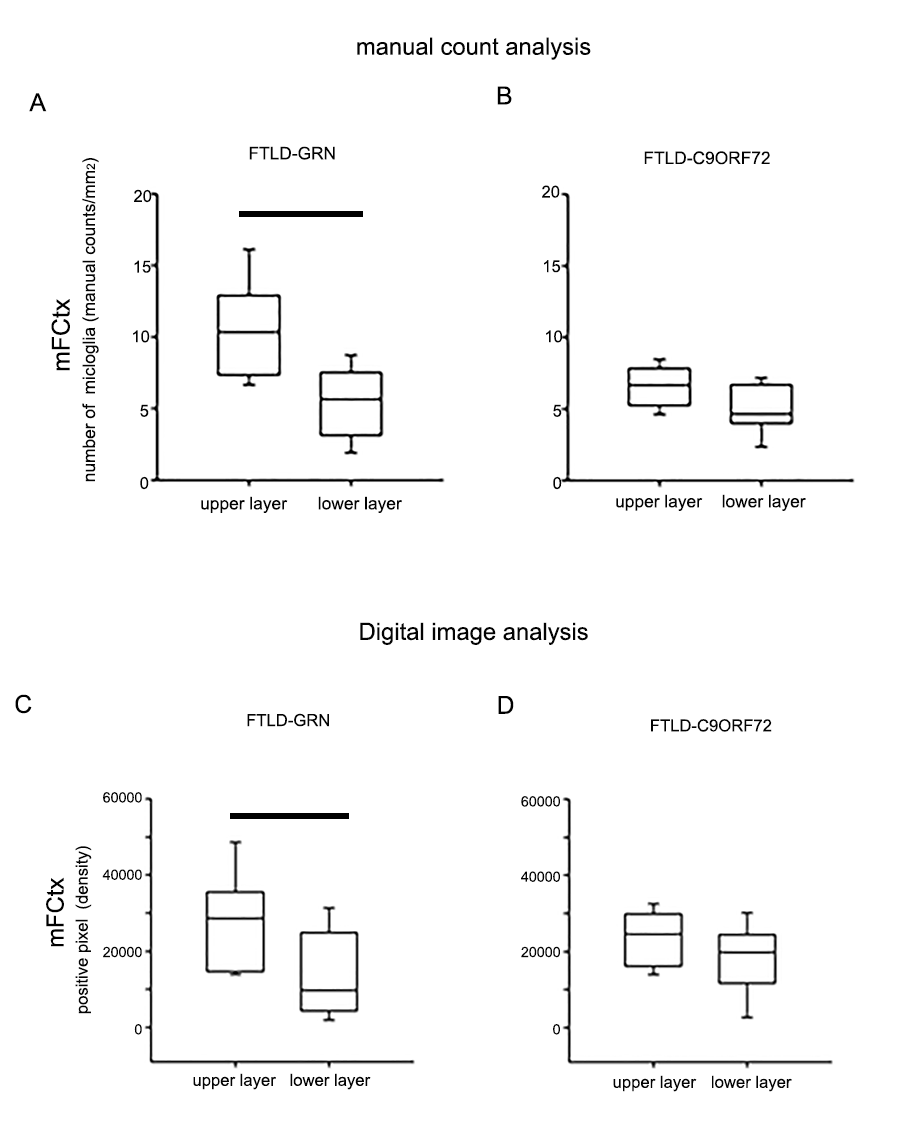

Supplement: Supplementary file 2 — Figure S1. Comparison of manual microglial counts to image analysis of IBA‐1 density. [file ACN3-6-1782-s001.tif]

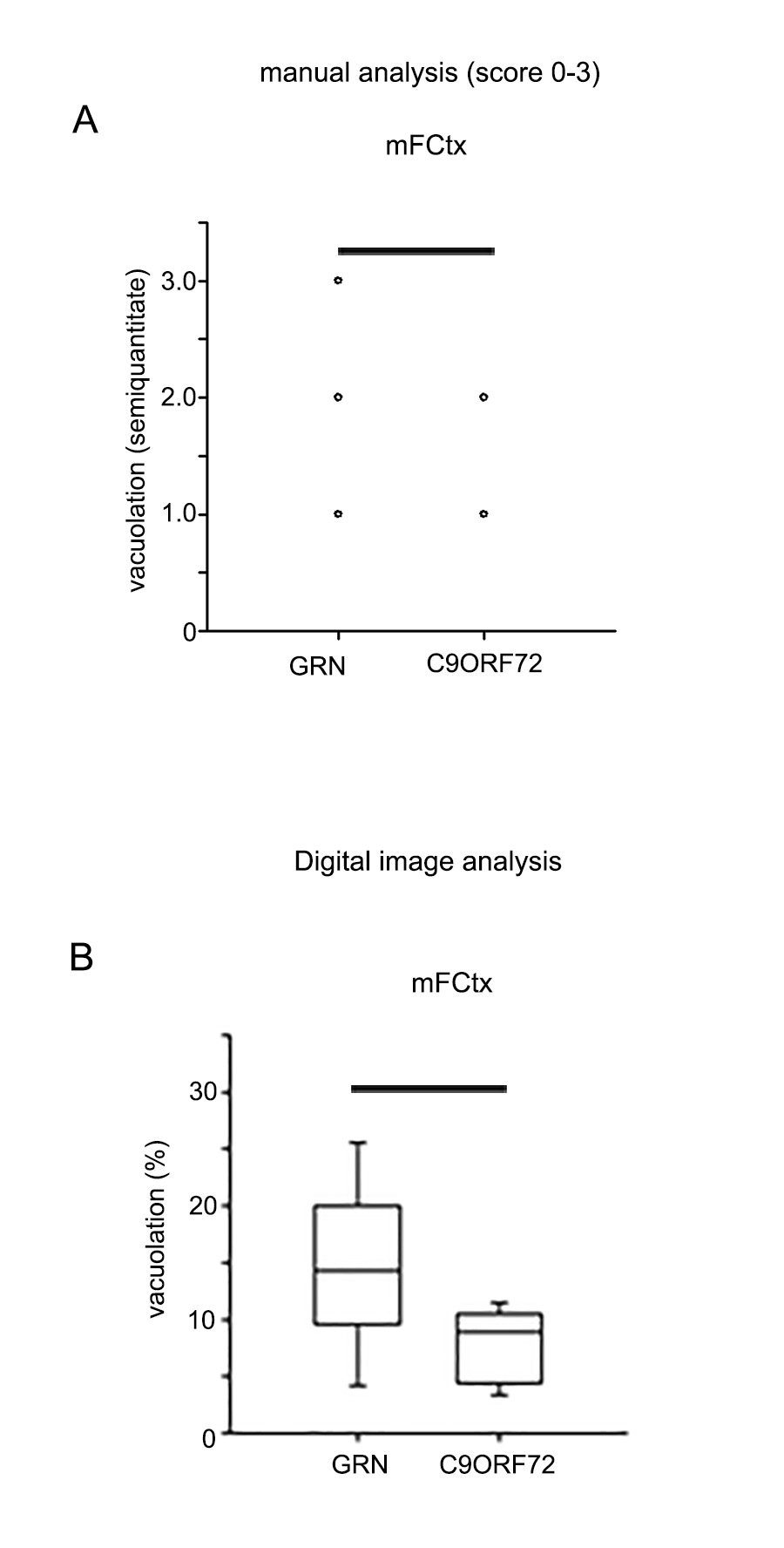

Supplement: Supplementary file 3 — Figure S2. Comparison of manual microvacuolation scores and vacuolation burden from image analysis. [file ACN3-6-1782-s002.tif]
